# Unexpected recombination at the polled locus in a horned Holstein calf from the mating of a homozygous polled sire and a heterozygous polled cow

**DOI:** 10.1111/age.13507

**Published:** 2025-01-08

**Authors:** Bertram Brenig

**Affiliations:** ^1^ Institute of Veterinary Medicine University of Göttingen Göttingen Germany

**Keywords:** BTA1, Celtic variant, Friesian variant, Holstein, horned, polled, recombination

## Abstract

In this study, I report an unexpected case of a Holstein calf that developed horns even though the sire was homozygous and the dam was heterozygous for polledness. After verifying and confirming the correct parentage, the parents and offspring were genotyped with the Illumina EuroG_MD BeadChip and the SNPs in the polled region on chromosome 1 were evaluated. In addition, the father was sequenced with next generation sequencing to identify possible, previously unknown variants. The deletion of two base pairs within the causative 80‐kb duplication described for the Friesian polled variant was verified by melting curve analysis and the 80‐kb duplication by droplet digital PCR. Analysis of all data showed that, as expected, the calf was heterozygous for all SNP positions flanking the 80‐kb duplication but was homozygous wild type in the 80‐kb duplication region and therefore carried horns. This is certainly a very rare case of a recombination within the highly conserved polled region, which on the one hand confirms that only the 80‐kb duplication is responsible for the expression of the Friesian polled variant, but on the other hand also shows that caution is required when interpreting the usual routine genotyping of the horn status based on linked single nucleotide polymorphisms in the polled region on chromosome 1. Based on the present case, it is recommended that, in addition to the evaluation of the SNP data of the BeadChip, an extended diagnosis with direct detection of the 2‐bp deletion (1:g.2629156_2629158delGT) should be carried out in any case when detecting the Friesian polled variant.

The natural occurrence of hornless cattle was already observed during the early domestication of cattle (Schafberg & Swalve, 2015). Since then, selection for hornlessness in cattle, also known as polledness, has been an important breeding goal (Scheper et al., 2016; Spurlock et al., 2014). Although horns are a normal trait in ruminants and may have behavioral and physiological functions, handling horned cattle can be dangerous not only for the owners but also for the animal itself or other animals (Knierim et al., 2015). For this reason, the search for the cause of genetic hornlessness began many decades ago. As early as 1993, a linkage analysis with microsatellites showed that the gene locus for genetic hornlessness is located on bovine chromosome 1 (BTA1) (Georges et al., 1993). With the development of DNA chip technology and efficient DNA sequencing methods, the actual genetic cause has finally been identified, although it is still completely unclear how these structural changes in a cow's genome lead to the development of hornlessness (Aldersey et al., 2020). To date, a total of four variants on BTA1 have been associated with polledness in different cattle breeds (*Bos taurus*, *Bos indicus*) (Randhawa et al., 2020). In Holstein cattle, two variants lead to the development of polledness, known as the Celtic (P_c_) and Friesian (P_f_) variants (Medugorac et al., 2012).

The Celtic variant is a 202‐bp indel (P_202ID_; 1:g.[2429327_2429336del; 2429109_2429320dupins, ARS‐UCD1.2]), while the Friesian variant is characterized by an 80‐kb duplication (P_80kbID_; 1:g.2629113_2709240dup, ARS‐UCD1.2). Within the 80‐kb duplication, a 2‐bp deletion (1:g.2629156_2629158delGT, ARS‐UCD1.2) can be used as a diagnostic marker (Aldersey et al., 2020; Medugorac et al., 2012). So far, these are the only known causative variants for polledness in Holstein cattle (Rothammer et al., 2014). As polledness is a dominant trait, the use of a homozygous polled parent is usually sufficient to breed polled offspring (Medugorac et al., 2012). In the present case, a sire tested as homozygous polled was mated to a cow tested as heterozygous, so that a polled offspring, either heterozygous or even homozygous, would have been expected in any case. However, the offspring developed horns.

In such a situation, the first assumption is that the parentage is probably false. Therefore, the parentage was checked using the recommended International Society for Animal Genetics microsatellite marker set for cattle before further investigation. The evaluation did not indicate that the parentage should be doubted (Table S1). To verify the polled status, the parents and the offspring were subjected to two additional tests. First, the 2‐bp deletion within the 80‐kb duplication associated with the Friesian polled variant was verified using a melting curve assay and second, droplet digital PCR was performed to determine the copy number of the 80‐kb duplication (Figure 1). Melting curve analysis was performed on a LightCycler 480 (Roche Diagnostics, Basel, Switzerland) using primers P80KIns(neu)f (5′‐GAAGTCGGTGGTCTGAAAGG‐3′), P80KIns(neu)r (5′‐TGTTCTGTGTGGGTTTGAGG‐3′), and P80KIns(neu)pWT (5′‐GTGTACGTGTGCACACACAGGGGA‐3′, labeled with 5′‐FAM, 3′‐BHQ1). The droplet digital PCR was performed with a QX200 Droplet Digital PCR System (BioRad, Feldkirchen, Germany). The primers mentioned above and, as a single‐copy gene control, the primers for the bovine *F2* gene (thrombin) bovThro.F (5′‐CCTGTCTGCTGAGACGCCG‐3′), bovThro.R (5′‐GTGGTAGAGTTGATTCTGGAATAGAAAGCAT‐3′), and BoThro_HEX (5′‐CCCCGCCACCCGCAGTGTCT‐3′, labeled with 3′‐HEX, 3′‐BHQ1) were used. The confidence intervals are 1.7–2.3 (p/p), 2.7–3.3 (P_f_/p), and 3.7–4.3 (P_f_/P_f_).

**FIGURE 1:**
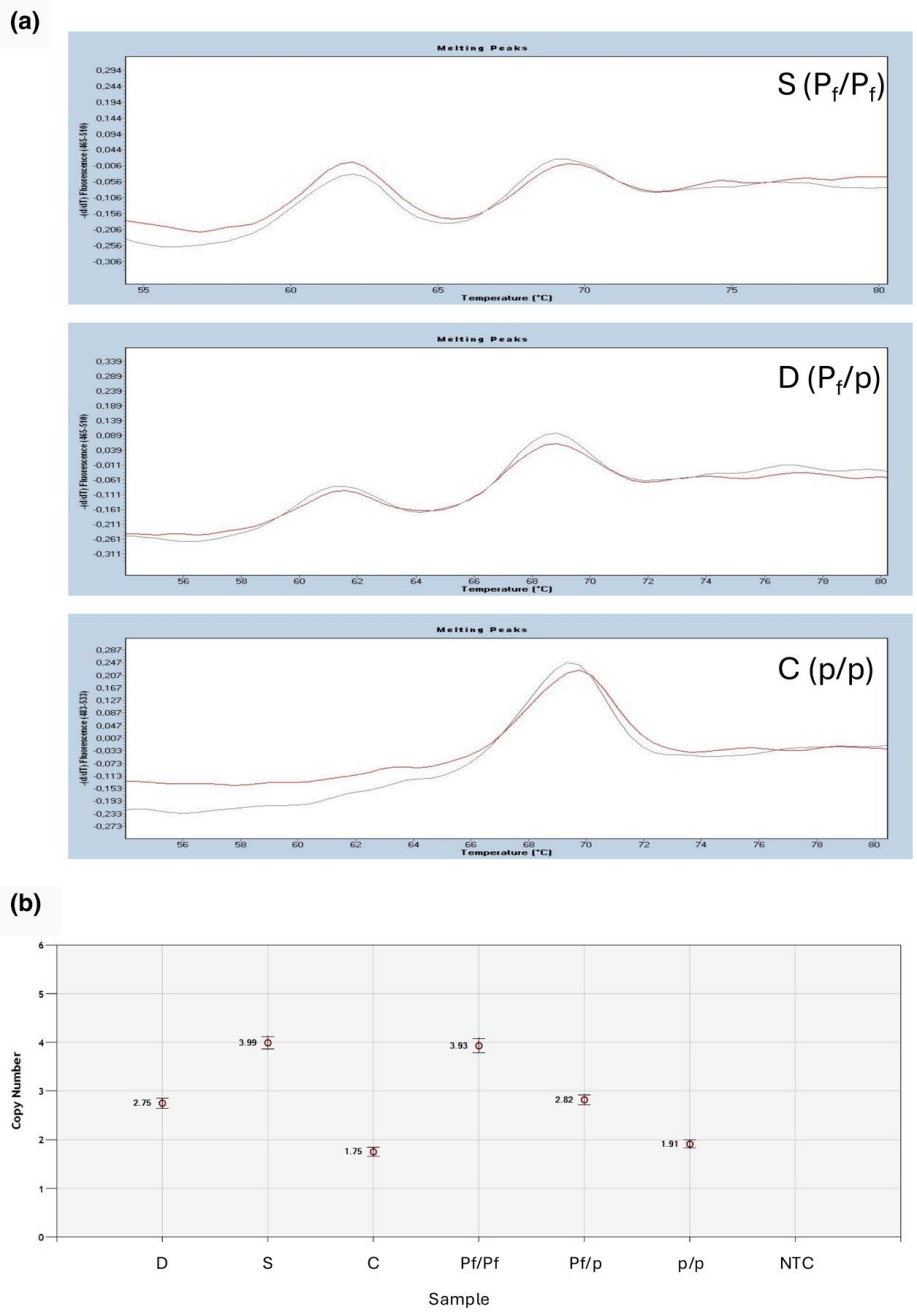
Melting curve analysis and droplet digital PCR of the Friesian polled variants. (a) The diagrams show the melting curves of the sire (S), dam (D) and calf (C) including the corresponding controls (gray lines: Controls; red lines: Sire, dam or calf). The polled genotypes are indicated (P_f_: Friesian polled allele; p: wild‐type allele). The presence of the Friesian polled allele (P_f_) is characterized by a peak at about 61°C. The melting peak of the wild‐type allele is at about 69°C. The height of the melting peaks reflects the number of Friesian polled alleles. The calf shows only the p allele, while the sire shows two P_f_ alleles and the dam one P_f_ allele. (b) In the digital droplet PCR analysis, the copy number of the 80‐kb DNA fragment is determined. The dam (D) shows three, the sire (S) four and the calf (C) two copies. Corresponding results of the controls (P_f_/P_f_, P_f_/p, pp) are shown. NTC, non‐template control.

Both genotyping tests confirmed that the sire was homozygous (P_f_/P_f_) and the dam heterozygous (P_f_/p) for the Friesian polled variant. The offspring, however, showed the homozygous wild type (p/p). To determine the genotypes of the flanking SNPs on BTA1, the DNA of the parents and the calf was genotyped using the Illumina EuroG_MD BeadChip. As shown in Table 1 the sire was homozygous and the dam and calf were heterozygous for all SNPs flanking the diagnostic 2‐bp deletion. However, the calf did not carry the 2‐bp deletion and showed the homozygous wild type alleles (II), which explains the development of the horns. In general, it cannot be excluded that such recombination can occur, but this event seems to be extremely rare, as not a single comparable result was detected in a period from 2017 to 2022, in which we tested approximately 6000 Holstein cattle for the Celtic and Friesian polled variants. This event supports the results found for the horned sire Rossi (p/p) (Rothammer et al., 2014). In this case, however, Rossi's parents were heterozygous polled (Lawn Boy P‐Red, P_f_/p) and homozygous horned (Renate, pp). Only recently, a Holstein trio was reported in which the sire was homozygous P_f_/P_f_ and the dam homozygous wild type (p/p) (Upadhyay et al., 2024). However, one offspring developed horns. By means of third generation sequencing it was shown that a recombination within the 80‐kb duplication had probably occurred in the sire, whereby the 5′ region in the offspring corresponded to the reference allele again. These results and the data presented here show that the evaluation of the Illumina BeadChip genotyping data can lead to conflicts and therefore great caution is required, especially if hornless status is derived solely from consideration of the chip genotyping data.

**TABLE 1:** Determination of SNP genotypes in the polled region on bovine chromosome 1 using the Illumina BeadChip (EuroG_MD).

BTA1 position (ARS_UCD2.0)	Calf (p/p)	Sire (P_f_/P_f_)	Dam (P_f_/p)
g.2372450C>T	CT	TT	CT
g.2372456A>G	AG	GG	AG
g.2377687G>A	AG	AA	AG
g.2378745C>T	CT	TT	CT
g.2395132 T>G	GT	GG	GT
g.2403929 T>C	CT	CC	CT
g.2429330G>T	GG	GG	GG
g.2429326G>A	CC	CC	CC
g.2486811 T>C	CT	CC	CT
g.2491161C>A	AC	AA	AC
g.2578598G>A	AG	AA	AG
g.2629156_2629158delGT^a^	II	DD	DI
g.2968717G>A	AG	AA	AG

^a^ The 2‐bp deletion within the 80‐kb duplication is indicated with II (wild type allele), DD (homozygous deletion), DI (heterozygous deletion).

## AUTHOR CONTRIBUTIONS

**Bertram Brenig:** Conceptualization; methodology; data curation; investigation; validation; formal analysis; visualization; project administration; resources; writing – original draft; writing – review and editing; supervision; funding acquisition; software.

## CONFLICT OF INTEREST STATEMENT

The author declares that he has no competing interests.

## Supporting information


Table S1.

## Data Availability

The genotype data are available at OSF (Doi: 10.17605/OSF.IO/F5UQH).
