# Supplementary material for: Unexpected recombination at the polled locus in a horned Holstein calf from the mating of a homozygous polled sire and a heterozygous polled cow
Source: Anim Genet. 2025 Jan 8;56(1):e13507. doi: 10.1111/age.13507 (PMC11707570; doi:10.1111/age.13507)
Supplement: Supplementary file 1 — Table S1. [file AGE-56-0-s001.docx]

**Supporting information**

**Table S1. Parentage control using ISAG recommended bovine microsatellite markers**

| **Microsatellite^a)^** | **Calf** | **Sire** | **Dam** |
| --- | --- | --- | --- |
| BM1818 | 266/270 | 266/270 | 266/266 |
| BM1824 | 182/182 | 182/188 | 178/182 |
| BM2113 | 127/137 | 125/127 | 127/137 |
| ETH3 | 117/127 | 117/127 | 117/129 |
| ETH10 | 219/225 | 225/225 | 219/223 |
| ETH225 | 150/150 | 150/150 | 148/150 |
| INRA023 | 210/214 | 210/214 | 206/210 |
| SPS115 | 248/248 | 248/248 | 248/252 |
| TGLA53 | 158/162 | 158/176 | 158/162 |
| TGLA122 | 149/183 | 143/149 | 163/183 |
| TGLA126 | 117/117 | 115/117 | 115/117 |
| TGLA227 | 89/89 | 89/91 | 89/97 |

a) ISAG recommended microsatellite markers for parentage control in cattle. Fragment lengths were adjusted to animal no. 13 from bovine comparison test 2005.
